# Longitudinal Study on Extended-Spectrum Beta-Lactamase-*E. coli* in Sentinel Mallard Ducks in an Important Baltic Stop-Over Site for Migratory Ducks in Germany

**DOI:** 10.3390/microorganisms10101968

**Published:** 2022-10-05

**Authors:** Sylvia Dreyer, Anja Globig, Lisa Bachmann, Anne K. Schütz, Katharina Schaufler, Timo Homeier-Bachmann

**Affiliations:** 1 Institute of International Animal Health/One Health, Friedrich-Loeffler-Institut, 17493 Greifswald, Germany; 2 Faculty of Agriculture and Food Science, University of Applied Science Neubrandenburg, 17033 Neubrandenburg, Germany; 3 Institute of Epidemiology, Friedrich-Loeffler-Institut, 17493 Greifswald, Germany; 4 Pharmaceutical Microbiology, Institute of Pharmacy, University of Greifswald, 17489 Greifswald, Germany; 5 Institute of Infection Medicine, Christian-Albrecht University Kiel and University Medical Center Schleswig-Holstein, 24105 Kiel, Germany

**Keywords:** antimicrobial resistance (AMR), ESBL-*E. coli*, birds, mallard ducks, sentinel

## Abstract

Antimicrobial resistance (AMR) is a serious global health threat with extended-spectrum beta-lactamase (ESBL)-producing Enterobacterales as the most critical ones. Studies on AMR in wild birds imply a possible dissemination function and indicate their potential role as sentinel animals. This study aimed to gain a deeper insight into the AMR burden of wild waterfowl by sampling semi-wild mallard ducks used as sentinels and to identify if AMR bacteria could be recommended to be added to the pathogens of public health risks to be screened for. In total, 376 cloacal and pooled fecal samples were collected from the sentinel plant over a period of two years. Samples were screened for ESBL-carrying *E. coli* and isolates found further analyzed using antimicrobial susceptibility testing and whole-genome sequencing. Over the sampling period, 4.26% (16/376) of the samples were positive for ESBL-producing *E. coli*. *Bla*_CTX-M-1_ and *bla*_CTX-M-32_ were the most abundant CTX-M types. Although none of the top global sequence types (ST) could be detected, poultry-derived ST115 and non-poultry-related STs were found and could be followed over time. The current study revealed low cases of ESBL-producing *E. coli* in semi-wild mallard ducks, which proves the suitability of sentinel surveillance for AMR detection in water-associated wildlife.

## 1. Introduction

The ability of pathogens to acquire, activate and exchange resistance genes is a steadily growing danger to public health and well known as antimicrobial resistance (AMR). Especially for bacterial pathogens, the increase in acquired resistances against antibiotics is of global concern. AMR is a natural and ancient ability, enabling, for example, bacteria to survive under adverse conditions [1]. Inappropriate antibiotic use, such as disregarding the length of the intake or application of the wrong dosage or due to wrong indication based on inappropriate or lacking prescription, can promote such conditions and lead to alterations in gut microbiota of humans and animals and select for resistant bacteria [2,3,4]. Together with partially metabolized antimicrobials, resistant bacteria are excreted into wastewater and may end up in surface waters and cropland via the sewage system, where they may enter the food chain, affect hygiene or spill-over to wild ecosystems [5,6]. A growing amount of data indicates that wildlife seems to play an important role in the spread of AMR [7,8,9]. Although wild animals do not generally like to be close to humans or human settlements, synanthropic animals may have more intensive contact with humans. This contact may play a role in the transmission of resistant bacteria, as already described for a wide variety of animal species, such as wild boar, roe deer, bats, marine mammals, pinnipeds, monkeys or wild birds [10,11,12,13,14,15,16,17,18,19,20,21,22,23], indicating that aquatic and terrestrial wildlife populations that are exposed to human residues have a higher prevalence of AMR bacteria than those living in more pristine areas [14,16,19,24,25,26,27]. Specifically, wild birds with frequent contact to human settlements may serve as vehicles to disseminate AMR bacteria or antimicrobial residues from anthropogenic sources over long distances [28,29,30,31]. However, the risk of AMR bacteria transmission between humans and wild or domestic animals, especially with a focus on such wild animals connecting all of them, is not clear [32,33].

Extended-spectrum beta-lactamase (ESBL)-producing Enterobacterales are considered highly important and, therefore, listed on the WHO’s “Global priority list of antibiotic-resistant bacteria to guide research, discovery and development of new antibiotics” [34]. ESBLs are mostly plasmid mediated and ESBL-producing bacteria show a high zoonotic potential [34,35]. Of particular concern is the emergence of multidrug-resistant (MDR) bacteria, which is defined as “non-susceptibility to at least one agent in three or more antimicrobial categories” [36,37]. Many antibiotics, including ß-lactams, are listed as “Veterinary Critically Important Antimicrobial Agents” by OIE (2021 OIE LIST OF ANTIMICROBIAL AGENTS OF VETERINARY IMPORTANCE) and as “Critically Important Antimicrobials” by the WHO (2018 WHO list of Critically Important Antimicrobials for Human Medicine). Obviously, ESBL-producing *E. coli* represents only one example of AMRB. However, the investigation of ESBL-producing *E. coli* is of such importance that they have been included in the WHO Tricycle Protocol as an indicator species using a One Health approach (https://www.who.int/publications/i/item/who-integrated-global-surveillance-on-esbl-producing-e.-coli-using-a-one-health-approach (accessed on 6 September 2022).

To detect the incidence of zoonotic pathogens of public and animal health concern transmitted by wild birds as early as possible, the Friedrich-Loeffler-Institute, which is the German Federal Research Institute of Animal Health, has been conducting sentinel surveillance for more than 15 years [38,39]. For this purpose, semi-wild mallard ducks, raised on farms, serve as sentinel ducks for infectious diseases, such as avian influenza. The plant is remotely located within the Greifswalder Bodden, which belongs to the 45% of protected German marine areas, shallow waters of the Vorpommersche Boddenlandschaft, which covers an area of 60,000 hectares and is an important stopover for annual bird migration, especially for many species of the order *Anseriformes* and *Charadriiformes*. Moreover, by providing water access, the plant allows ducks to be in direct contact with wild birds, such as (migratory) waterfowl species or other wild birds small enough to pass through the grid [38], allowing for free pathogen exchange, such as AMR bacterial species among the animals. To our knowledge, nothing is known about the role of semi-wild birds as sentinels to detect AMR in wildlife. In the present study, these sentinel ducks with contact to wild birds were sampled regularly to investigate the appearance of ESBL-*E. coli* as an indicator for AMRB over a two-year period. Altogether, this study provides unique insights into a semi-wildlife AMR profile, which could also prospectively serve as a model for AMR detection at the human/livestock/wildlife/environmental interface.

## 2. Materials and Methods

### 2.1. Study Design

Sentinel ducks were sampled by taking individual cloacal swabs every two to four weeks on a regular basis. If available, pooled fecal samples from sentinels and other animals that sporadically entered the facility (e.g., rats) were collected within the plant area. Since the animals were also screened for avian influenza, the occurrence of highly pathogenic avian influenza resulted in the elimination of the whole group. Therefore, this study consisted of two different mallard duck cohorts. Cohort 1 consisted of 15 individuals and was sampled between May 2020 and January 2021. Cohort 2 consisted of 10 individuals that arrived in June 2021 and were joined by an additional 5 animals in July 2021. Thus, the total number in cohort 2 as of July 2021 was 15 animals sampled until March 2022. 

### 2.2. Ethical Approval

Animal experiments were approved by the State Office of Agriculture, Food safety, and Fishery in Mecklenburg-Western Pomerania under the registration number LALLF 7221.3-2-006/19 including the approval and control of the local veterinary authority of Greifswald municipality to keep mallard ducks for scientific purposes.

### 2.3. Bacterial Isolation

Swabs from cloacal and fecal samples were incubated overnight in LB medium (Lennox) (Carl Roth, Karlsruhe, Germany) containing 2 µg/mL cefotaxime (VWR International GmbH, Darmstadt, Germany) at 37 °C while shaking at 200 rpm and subsequently cultivated on CHROMagar Orientation plates (MAST Diagnostica, Reinfeld, Germany) supplemented with 2 µg/mL cefotaxime at 37 °C. Putative cefotaxime-resistant *E. coli* colonies were identified based on colony morphology (pinkish colonies) and subjected to further cultivation until pure cultures were obtained. Pure cultures were used for further verification and characterization.

### 2.4. Antimicrobial Susceptibility Testing (AST) Using VITEK 2

For further phenotypical identification of the ESBL-*E. coli* isolates, samples were cultivated on Columbia blood agar plates containing 5% sheep blood (Becton Dickinson GmbH, Heidelberg, Germany) and handed to the VITEK 2 analysis automated system (bioMérieux, Marcy l’Etoile, France). The assay was conducted according to the manufacturer’s instructions. The assay was performed using software version 9.02 and AST-N389 cards. All results were interpreted according to EUCAST breakpoints and guidelines.

### 2.5. Whole-Genome Sequencing (WGS) and Analysis

WGS was performed for the overall 21 ESBL-suspect *E. coli* isolates. For DNA extraction the MasterPure™ DNA Purification Kit for Blood, Version II (Lucigen, Middleton, WI, USA) was used. DNA yields were quantified using QuBit 4 fluorometer (Thermofisher Scientific, Waltham, MA, USA). DNA samples were shipped to Microbial Genome Sequencing Center (MiGS, Pittsburgh, PA, USA). Sample libraries were prepared using the Illumina DNA Prep kit and IDT 10 bp UDI indices and sequenced on an Illumina NextSeq 2000, producing 2 × 151 bp reads. Demultiplexing, quality control and adapter trimming were performed with BCL Convert v3.9.3 (Illumina, Inc., San Diego, CA, USA; https://support-docs.illumina.com/SW/BCL_Convert/Content/SW/FrontPages/BCL_Convert.htm (accessed on 1 August 2022).

The sequence analysis has been described previously [5,10]. In brief: BBDuk from BBTools v. 38.89 (http://sourceforge.net/projects/bbmap/ accessed on 1 May 2022) was used for adapter trimming, contaminant filtering, and quality trimming. FastQC v. 0.11.9 (http://www.bioinformatics.babraham.ac.uk/projects/fastqc/ accessed on 1 May 2022) was used for quality controlling of all reads (trimmed reads and raw reads). The shovill v. 1.1.0 assembly pipeline (https://github.com/tseemann/shovill accessed on 1 May 2022) in combination with SPAdes v. 3.15.0 [40] was used for de novo genome assemblies. Assemblies were analyzed for multi-locus sequence type (MLST) determination and antibiotic resistance gene detection using the tools mlst v. 2.19.0 (https://github.com/tseemann/mlst accessed on 1 May 2022) and ABRicate v. 1.0.0 (https://github.com/tseemann/abricate accessed on 1 May 2022), respectively. Third-party databases (e.g., ResFinder [41], PlasmidFinder [42], ARG-ANNOT [43], and PubMLST [44] accessed on 1 May 2022) were used for the analyses of both tools.

## 3. Results

### 3.1. Bacterial Isolation

**Figure 1:**
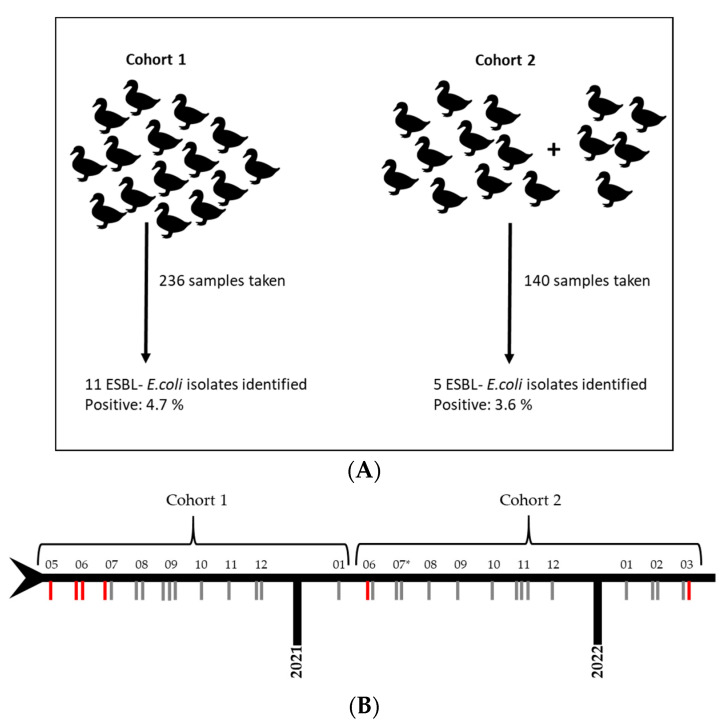
Schematic illustration of the sampling procedure and positive cases. (**A**) Two cohorts were sampled regularly. Cloacal swabs and pooled fecal samples were taken. Samples were investigated for extended-spectrum beta-lactamase (ESBL)-*E. coli* presence by bacterial cultivation. *E. coli* identification was additionally confirmed by whole genome sequencing. (**B**) Timeline of the sampling frequency (gray lines) starting in May 2020 until March 2022. Months (indicated as numbers) without sampling activity are not shown. Positive ESBL-*E. coli* results are shown in their order of appearance and highlighted as red lines. * indicates arrival of additional 5 animals in cohort 2.

Samples from two mallard duck cohorts were collected between May 2020 and March 2022 every two to four weeks and analyzed for the presence of ESBL-producing *E. coli*. In cohort 1, a total of 11 putative ESBL-producing *E. coli* isolates from 236 samples (incl. 11 from fecal samples) was found. Of these, 9 isolates originated from cloacal swabs and 2 from feces. In cohort 2, five putative ESBL-*E. coli* isolates from 147 samples (incl. 7 from feces) were obtained, 4 from cloacal swabs and 1 from a fecal swab. Thus, 4.7% of the samples were positive for ESBL-producing *E. coli* for cohort 1 and 3.6% for cohort 2 (see Figure 1A and Table 1). Displayed in a timeline of results, an increased occurrence of positive results within cohort 1 compared to cohort 2 is apparent (see Figure 1B). It also shows the disappearance of cases two months after the ducks arrived at the facility, although this again differed between the cohorts. Interestingly, after 9 months of regular testing with negative results in cohort 2, ESBL-*E. coli* isolates were detected again in that cohort (see Figure 1B).

### 3.2. Antimicrobial Susceptibility Testing (AST)

All 16 isolates were resistant to the beta-lactam antibiotics amoxicillin, in the absence and presence of clavulanic acid, ampicillin, cefalexin, cefotaxime, ceftazidime, and cefepime. In addition, two isolates from cohort 1 (numbers 285 and 275) showed resistance to other antibiotics as well, thus, fulfilling the definition as MDR (12.5%, 2/16). Both 275 and 285 exhibited resistance against the combination of trimethoprim/sulfamethoxazole, whereas 285 completed its resistance profile by resistance against ciprofloxacin. All isolates were sensitive to imipenem, meropenem, amikacin, gentamicin, tobramycin, tigecycline, fosfomycin, and colistin. Details are given in Table 2.

### 3.3. Whole-Genome Sequence (WGS) Analysis

**Figure 2:**
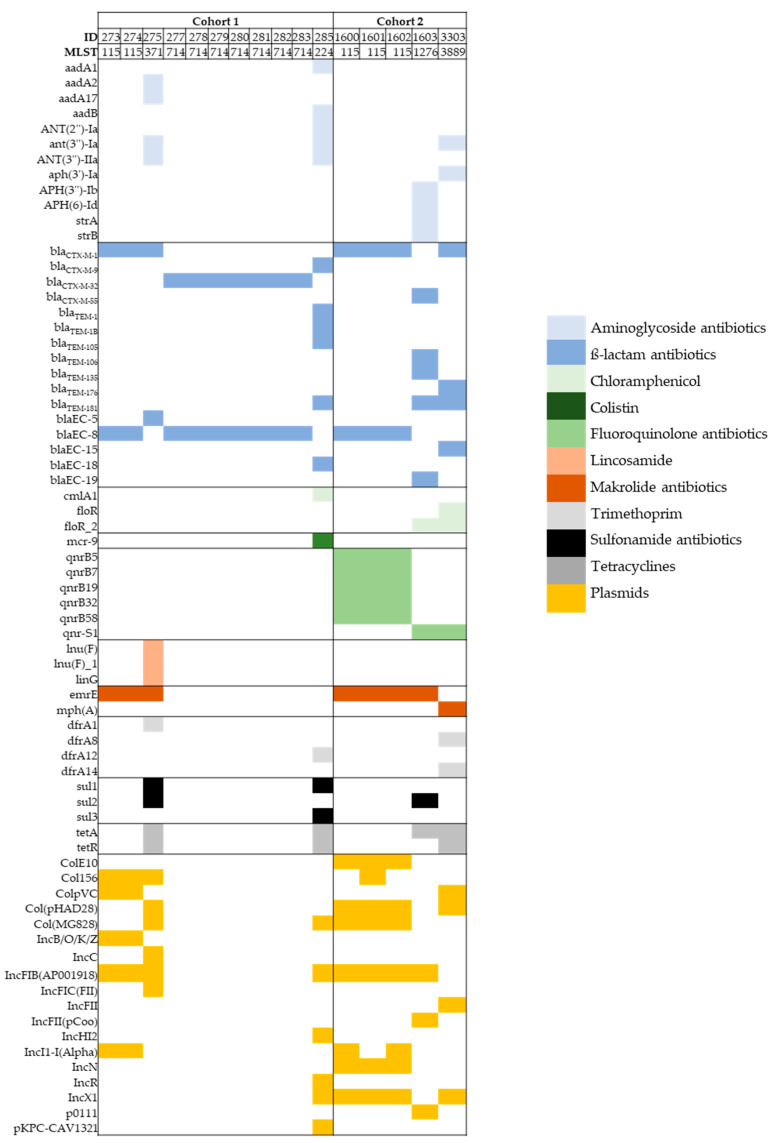
Presentation of genotypic data. Genetic data represent selected results from ABRicate (ARG-ANNOT, resfinder, megares, card, ncbi, plasmidfinder). The header lists the respective isolate number (ID) with the related multi-locus sequence type (MLST) (PubMLST). Resistance genes found in all isolates are not shown in the figure.

To gain more knowledge on each of the 16 isolates at the molecular level, WGS was applied. MLST analysis revealed six different ST. ST714 was most commonly detected (*n* = 43.75%, 7/16), followed by ST115 (*n* = 31.25%, 5/16). ST371, ST224, ST1276 and ST3889, each found only once. Interestingly, while ST115 appeared in both cohorts, ST714 could only be detected in cohort 1 and only once in a sampling round. Genes encode for various proteins associated with antibiotic resistance against cefotaxime and third-generation cephalosporin, e.g., *bla*_CTX-M-1_, and trimethoprim, e.g., *dfrA12*, were detected (see Figure 2). In addition, genes encoding for resistance to multiple drugs and antimicrobial peptides were found widely (e.g., *marA*, *tolC*, *pmrE*, *ugd*). Resistance to the following antimicrobial classes (and AMR genes) were detected in all isolates: aminocoumarine antibiotics (*mdtA*, *mdtB*, *mdtC*), aminoglycoside antibiotics (*acrD*, *kdpE*), beta-lactame antibiotics (*ampC1*, *ampC2, ampH, OmpA*), fluoroquinolones (*emrA, emrB, emrR, mdtH*), fosfomycine antibiotics (*mdtG*), nitroimidazole antibiotics (*msbA*), polypeptides (*bacA*) and tetracyclines (*emrK, emrY*) (and were, therefore, not included in Figure 2). A number of plasmids (*n* = 18) was detected throughout the cohorts. Except for isolates identified with ST714, all isolates carried three to seven plasmids with IncFIB (AP001918) as the most common one with 50% (8/16), followed by Col (pHAD28), Col (MG828) and IncX1 with 31.25% (5/16), respectively, followed by Col156 and IncI1with 25% (4/16) each, followed by ColE10, ColpVC and IncN with 18.75% (3/16) each, followed by IncB/O/K/Z with 12.5% (2/16). Plasmids identified as IncC, IncFIC (FII), IncFII, Inc (pCoo), IncHI2, IncR, p0111 and pKPC-CAV1321 were detected only once (see Figure 2).

## 4. Discussion

In the present study, the rate of ESBL-*E. coli* in semi-wild mallard ducks, kept as sentinel ducks in a sentinel surveillance approach, was investigated over a period of two years. The study revealed that 4.26% of all samples investigated were positive for ESBL-*E. coli*. It was also found that all isolates were positive for AmpC-encoding genes. However, this finding was not in the scope of our study and was, therefore, not investigated further.

Among wild animals, a special role in spreading AMR has been proposed for wild birds as they are able to migrate over vast distances [28,29,45,46,47,48]. The studies range from investigations of very remote areas with scarce human influence, such as Antarctica or Alaska [26,49], to areas with high human influence [50], such as poultry farms [51,52,53,54]. While in the study from Rabbia et al. [55] no AMR bacteria could be identified in fecal samples from wild birds, positive results from environmental samples close to human activities, such as waste water treatment plants, showed a potential risk. A study by Ramey et al. [27] indicated a very low prevalence of antimicrobial-resistant *E. coli* and even no positive results on ESBL-*E. coli* in migratory wild birds in remote Alaska. In contrast, in areas with high human influence (e.g., in close proximity to settlements), detection rates of AMR can be high. Wyrsch et al. [12] investigated gull chicken in Australia. Among others, they reported a reduced susceptibility to cefotaxime in 64% of the isolates (273/425). Other studies investigating wild birds confirmed a higher ESBL-*E. coli* rate in urban settings compared to rural areas, as in a study from Loucif et al. [56]. They found a prevalence of 12% ESBL-*E. coli* in pigeon in Algeria. The different results between the two studies could be explained by the different methods used. Loucif et al. used pooled fecal samples, whereas Wyrsch et al. investigated individual samples. Although both studies started with an enrichment step before cultivation, Loucif et al. used selective enrichment (vancomycin + another antibiotic), while Wyrsch et al. did not use vancomycin at all. 

Studies on poultry farms usually reflect a higher prevalence. Vounba et al. [53], reported a relatively low prevalence of ESBL/AmpC-*E. coli* with 3.9% but comparable to our results. In a study by Falgenhauer et al. [51], investigating ESBL-*E. coli* in poultry in Ghana, a prevalence of 29% was detected. Since these findings indicate a rather low burden in the poultry sector, other studies investigating ESBL-*E. coli* identified a much higher prevalence of almost 92% in a study in Spain [57] and 75% in a study in Egypt [52], which reflect the dramatic situation of resistant strains in the food-producing sector. Depending on the husbandry system, poultry that is kept outside or ranges freely might come in contact with wild birds, which might result in an exchange of pathogens between the two populations. In addition, feeding of poultry might attract wild birds, which further increases the interface and provides another opportunity for pathogen transmission, such as avian influenza. In summary, ESBL-*E. coli* are present in (wild) birds around the globe and in each setting. Results on ESBL-*E. coli* in birds vary widely and depend on species, behavior and location, with migratory wild birds harboring, usually, lower loads of resistant bacteria than resident birds [45]. 

Almost all studies on AMR in wildlife indicate that birds (especially migratory birds) could serve as vectors for AMR and they are, thus, fondly referred to as indicators for the presence of AMR in wildlife. However, to our knowledge, sentinel surveillance has never been conducted for AMR. Here, we present a detection rate of 4.26% ESBL-*E. coli* in sentinel ducks, which rather fits the range of the results from the abovementioned wild bird literature. Further analysis on a phenotypical and molecular level in this study revealed more information.

The comparison of genotypes with phenotypes showed a high concordance of results with the class of ß-lactam antibiotics, which was to be expected due to the pre-selection step with the antibiotic cefotaxime during enrichment of the isolates. Two isolates also showed resistance genes to trimethoprim, thus, confirming our phenotypic results. Of note, one isolate was found to be phenotypically positive for resistance to fluoroquinolone antibiotics (ciprofloxacin), which was not confirmed on a molecular level, indicating chromosomal point mutations as a cause. However, investigating point mutations causing AMR phenotypes and association between AMR genes and plasmids were beyond the scope of this study. Since our Vitek analysis did not cover all antibiotic classes, we cannot exclude more concordance between the two data sets. When including sequence types as well, the data indicate that all isolates, which show a different ST than 115 or 714 (namely 275, 285, 1603, 3303), seem to carry more additional resistance genes than others, thus, indicating a higher resistance potential.

Regarding the classification of where the animals potentially acquired ESBL-*E. coli*, the following picture emerges from the two cohorts. Duck isolates from cohort 1, taken within a period of two weeks after arrival, might be derived from the farm of origin as they shared the same sequence type ST115, which is well connected with poultry [58] as well as the CTX-M-1 type, which is the major ESBL enzyme in poultry [59]. In addition, a review of records in the Enterobase database [60] revealed that for ST115, the poultry sector was most frequently reported as the origin of isolates (76.7%). Furthermore, two isolates carried a CTX-M-1-IncI1 plasmid, described to be mainly poultry derived [61]. Interestingly, two weeks later, one of the ducks was still positive but now showed a different sequence type (ST224), which has been described to belong to a potentially high pathogenic global lineage identified in wild birds [62], humans [63], chickens [64] and wastewater samples [65], indicating an external entry potentially by wild birds. Moreover, this strain was the only ST in our study that carried a colistin-resistance gene (*mcr-9*). Although *mcr-9* is the youngest member of the *mcr* family [66], it is already the most disseminated variant together with *mcr-1* [67]. However, in our study the isolate did not exhibit phenotypic resistance to colistin. This finding is in concordance with a study from Tyson et al. [68], who discussed that the *mcr-9* gene carrier does not necessarily show a colistin-resistance phenotype, which is also true for other *mcr* variants. Although this indicates no benefit for the carrier, *mcr-9* is mainly found in company with other resistance genes, allowing for a co-selection in the presence of other antimicrobials [68]. Furthermore, in a study from Kieffer et al. [69], they detected *mcr-9* on an IncHI2 plasmid and showed that the presence of the gene alone does not lead to clinical resistance to colistin, but only when induced by regulatory genes. Based on their findings, they indicate a silent spread of *mcr-9*-mediated colistin resistance. The results found for ST224 were confirmed by studies from Algeria, which performed studies on chicken and pigeons [56,70]. The occurrence of ST224 in different hosts and under different conditions may be a reason why it could have displaced the previously detected ST115. However, CTX-M-9-type ESBL-*E. coli* seems to be limited in their zoonotic potential, as concluded by Yamasaki et al. [71], although they belong to the most widespread clones [72]. In contrast, the isolates derived from samples taken from the sentinel ducks in July 2020 differed strongly from the first isolates. None of the animals were tested positive before and the sequence type (ST714) has not been present in the sentinel system before either. Although a replacement of one ST by another more successful one has been described, as for the current rapidly emerging ST1193, replacing even the most frequent clone ST131 at some regions [73], it cannot be excluded that the emergence of ST714 was missed at an earlier stage, although the whole cohort was sampled every two weeks. So far, there are only a few studies available. However, in a study from Matsukawa et al. 2019 [74], they isolated ST714 from patients suffering from community acquired acute urinary tract infection, which was also reported by the study of Hertz et al. [75]. On the other hand, Haenni et al. [76] identified ST714 in Enterobacteriaceae in wild birds. Since sampling always took place in a full body suit, contamination while sampling seems rather unlikely. In addition, there are, so far, only reports from outside of Germany, suggesting an entry from wild animals. This assumption is supported by the data sets of the Enterobase database [60]. There are currently 32 entries for ST714, 19 of which originate from the environment or wildlife sector. A core single-nucleotide polymorphism analysis [77] of the available Illumina read data revealed that the next most similar isolate, however, originates from the human sector and, like our ST714 isolates, also carries a CTX-M-32 [78]. This finding underlines the zoonotic nature of these isolates. Furthermore, no plasmids could be identified for ST714 isolates when applying BLAST search or using plasmidfinder, which indicates rather chromosomal encoding of *bla* genes than plasmid encoded, as already observed in a study by Guenther et al. [23]. In addition, the Enterobase entry of the next most similar isolate showed the same picture and also the absence of other resistance genes, separate from *bla*. The discovery of another isolate of non-duck origin with a so-far-undetected sequence type (ST371) in this sentinel plant indicates the presence of a duck–wildlife interface within the plant and the possibility of exchange. However, studies on ST371 showing findings in mammals are rather rare [79], indicating a strain preferentially related to birds [80,81,82]. Interestingly, for some reason, such as age of the animals or environmental conditions, as indicated by a study from Hess et al. [83], this strain did not seem to be particularly well adapted due to the lack of establishment, which seems to be in contrast to the ST714 isolates, which were able to spread better, albeit transiently. In summary, 2/11 isolates most likely originated from the farm they were bred, while the other isolates could have originated from wild animals, as described above. 

Cohort 2 isolates detected upon arrival were probably from the farm of origin as well. Again, the poultry-related ST115 [58] could be identified but failed to “establish” in the sentinel population. Another finding was ST1276, which was also associated with poultry farming [82,84,85,86]. In addition, it has been reported that the carriage of CTX-M-55 plasmid in ESBL-*E. coli* might be associated with spread to humans via food [69]. Studies reporting findings of ST3889 are scarce. A study from Kim et al., investigating retail meat from different countries sold in South Korea, found ST3889 in pork meat from Germany [87]. In another study, from Wang et al., ST3889 could be identified from feces samples of diarrheic yaks living under semi-intensive farm conditions in Korea [88]. Based on the near absence of data and the lack of interpretation of the finding of ST3889 in these studies, this may be a strain acquired from wildlife. The long-time lag between entry of the new ducks in July 2021 and identification of ST3889 isolate in March 2022 is another fact supporting this assumption, although it cannot be excluded that the emergence of ST3889 was missed at an earlier stage. Thus, most of the isolates from this cohort seemed to be introduced by animal husbandry, whereas one isolate indicates an entry by wild animals. However, none of the top global STs, such as ST131, ST410, ST10 or ST648, could be detected [89].

Sampling was performed in full body suits and human–duck contact was reduced to a minimum while feeding. However, since human samples were not part of the study, contamination is highly unlikely, but cannot be completely excluded. Although sampling followed a regular scheme, some sampling events were omitted, mainly because the operators were not available due to COVID-19 restrictions, which may have led to some strains being unnoticed. Since only one colony showing an ESBL phenotype was selected for WGS analysis per plate, the other clones exhibiting a different ST might have been overlooked. Further studies, including human, environmental and climate data sampling, as well as farm sampling, would enable us to gain knowledge on the source of infection, the chronological trend and the identification of drivers.

## 5. Conclusions

AMR is a growing public health problem around the globe, which affects human and animal health. Migratory birds have been indicated as possible carriers of AMR, even over long distances, but studies on the prevalence of AMR in wildlife are still scarce. Wild birds, in particular those found in the vicinity of human activities, have been attributed a possible sentinel function. Here, we show that sentinel surveillance is suitable for AMR detection in wildlife. However, it is important to distinguish them from introduced isolates from (poultry) livestock.

## Figures and Tables

**Table 1:** Sampling details comprising sample date and sample type. Positive results are connected to their isolate numbers and sample source.

Cohort	Year	Sampling Date	Positives/Number of Samples Taken	Sample Type	Isolate No. (Origin of Sample)
1	2020	27 May	1/15	C	274 (11)
		10 June	1/15	C	273 (5)
			1	F	275 (pooled, non-duck)
		25 June	1/15	C	285 (5)
		8 July	6/15	C	277–282 (1–3, 9, 10, 13)
			1	F	283 (pooled)
		22 July	0/15	C	
		5 August	0/15	C	
		18 August	0/15	C	
		1 September	0/15	C	
		16 September	0/15	C	
		30 September	0/15	C	
		13 October	0/15	C	
		11 November	0/15	C	
		2 December	0/15	C	
		22 December	0/15	C	
	2021	19 January	0/15	C	
2	2021	3 June	3/10	C	1600–1602 (1, 2, 10)
			1	F	1603 * (pooled)
		17 June	0/10	C	
		8 July	0/9	C	
		30 July	0/9	C	
		26 August	0/10	C	
		14 September	0/12	C	
		26 October	0/7	C	
		8/9 November	0/5	C	
		17 November	0/7	C	
		23 November	0/6	C	
		15 December	0/8	C	
	2022	14 January	0/5	C	
		3 February	0/7	C	
		24 February	0/8	C	
		9 March	0/4	C	
		March	1/13	C	3303 (12)

* from transport box, sample type (C = cloacal, F = fecal), origin of sample indicates the IDs of the sampled individuals with positive results in brackets unless indicated otherwise, non-duck = putative mammal origin.

**Table 2:** Phenotypic resistance profiles of extended-spectrum beta-lactamase (ESBL)-*E. coli* isolates obtained from cloacal samples of semi-wild mallard ducks and pooled fecal samples.

Cohort	Designation	AMO	AMP	AMO/CA	CFL	CTX	CAZ	CFZ/TAZ	FEP	IMI	MEM	AMI	GEN	TOB	CIP	TGC	FOS	COL	TMP/SMX	ESBL	MDR
1	273																			X	
	274																			X	
	275																			X	X
	277																			X	
	278																			X	
	279																			X	
	280																			X	
	281																			X	
	282																			X	
	283																			X	
	285																			X	X
2	1600																			X	
	1601																			X	
	1602																			X	
	1603																			X	
	3303																			X	

Legend: AMO = amoxicillin; AMP = ampicillin; AMO/CA = amoxicillin/clavulanic acid; CFL = cefalexin; CTX = cefotaxime; CAZ = ceftazidime; CFZ/TAZ = ceftolozane/tazobactam; FEP = cefepime; IMI = imipenem; MEM = meropenem, AMI = amikacin; GEN = gentamicin; TOB = tobramycin; CIP = ciprofloxacin; TGC = tigecycline, FOS = fosfomycin; COL = colistin; TMP/SMX = trimethoprim/sulfamethoxazole. Resistant (grey boxes), sensitive (blank boxes).

## Data Availability

Data for this study were deposited in the European Nucleotide Archive (ENA) at EMBL-EBI under accession number PRJEB54078 (https://www.ebi.ac.uk/ena/browser/view/PRJEB54078 (updated and published on 4 October 2022).
